# Reproductive organ on-a-chip technologies and assessments of the fetal-maternal interface

**DOI:** 10.3389/frlct.2024.1449303

**Published:** 2024-09-05

**Authors:** Hannah A. Richards, Alison J. Eastman, Dusty R. Miller, David E. Cliffel

**Affiliations:** 1 Department of Chemistry, Vanderbilt University, Nashville, TN, United States,; 2 Department of Obstetrics and Gynecology, Vanderbilt University Medical Center, Nashville, TN, United States,; 3 Vanderbilt Institute for Integrative Biosystems Research and Education, Vanderbilt University, Nashville, TN, United States

**Keywords:** organ-on-chip, fetal membrane on-a-chip, microfluidic, fetal membranes, placenta, female reproductive tract

## Abstract

In this review, we discuss recent reproductive organ-on-a-chip (OoC) experiments that encompass multiple target areas of investigation, including model fabrication strategies, transport mechanisms, and immunology. We highlight fetal membrane and placental biology, OoC history and background, and the designs of reproductive OoC platforms. Reproductive OoC designs include fetal membrane models such as the Fetal Membrane-on-a-chip (FMOC) and others, placental models such as the placenta on-a-chip, and full reproductive tract models such as EVATAR. Diverse fabrication strategies and the integration of multiple model materials are explored. OoC samples can be analyzed with many analytical techniques, including mass spectrometry, fluorescence microscopy, ELISAs, impedance spectroscopy, and electrochemical techniques. The future of reproductive OoC models is a promising technology for advancing preterm birth (PTB) research, pharmacology studies, and fertility technologies.

## 1 Introduction

Every year approximately 15 million infants are born preterm—before completing 37 weeks of gestation—making preterm birth (PTB) and PTB-related complications the leading cause of death among children worldwide (Walani, 2020). If the trend continues, it is estimated 4.4 million children under the age of 5 years old will die in 2030 from PTB-related complications (Liu et al., 2015). Current PTB treatments and remedies remain insufficient as reproductive studies involving pregnant women and fetuses are ethically and legally hindered, thus the need for an alternative system remains. A deeper understanding of the human fetal membranes and the placenta can be gained from the use of 3D organotypic devices, known as organ on-a-chip (OoC) technologies, that aim to duplicate the physiological and cellular context of biological tissues in a small model (Wikswo, 2014; Young and Huh, 2021). In this review, we explore fetal membrane and placental biology, background and OoC history, and recent reproductive OoC model investigations. We give an overview of the questions that researchers are asking, the designs of these models, and how they integrate immunology, fluidics, and synthetic materials into these systems. To conclude, considerations for drug testing and future reproductive advancements are discussed.

Reproductive OoC technologies are an emerging method for examining fetal membrane and placental biology. Fetal membrane and placental development and their supportive functions are key components in fetal maturation. This development can be displayed and manipulated in OoC platforms. The fetal membranes and the placenta interact through the fetal-maternal interfaces, the amniochorion-decidua parietalis and the placenta-decidua basalis, making study of the two organs in a single model advantageous. Likewise, while the reproductive organs are interconnected at the fetal-maternal interfaces, the placenta and the fetal membranes are two different organs with distinct activities and compartments. The functional and biological differentiation of the placenta and fetal membranes makes individual study of the two organ models viable.

OoC platforms, including the fetal membrane on-a-chip (FMOC), placenta on-a-chip, and multi-reproductive organs ona-chip, are designed to mimic the cellular environment *in vivo*. These unique designs can be achieved with various fabrication materials with distinct oxygen permeability and absorption properties, allowing each model design to be experimentally optimized. Common materials include polymethylmethacrylate (PMMA), the highly oxygen-permeable polydimethylsiloxane (PDMS), low molecular-absorption Flexdym (Lachaux et al., 2017), and the low oxygen-permeable cyclic olefin copolymer (COC) (Ochs et al., 2014). The device typically consists of an etched, molded, or polymer material sandwiched onto a solid plate to form a microfluidic channel. Cell cultures can grow within the 3D chamber model with dynamic media perfusion mimicking the sheer and mechanical stresses experienced *in vivo*, proving 3D culture advantageous to previous 2D cell culture studies. Many OoC designs are limited to two or three microfluidic chambers, yet recent developments have innovated platforms to consist of six or more chambers for the incorporation of multiple organ systems (Srivastava et al., 2024). The design of multiple chambers within one model incorporates multiple cell layers and cell types, which is an important detail for the investigation of transport mechanisms and immune responses across cell layers.

OoC models can be paired with a wide range of analytical instrumentation to identify immune responses and to monitor transport mechanisms. Analytical methods can be as direct as fluorescence microscopy and enzyme-linked immunosorbent assays (ELISA) to more indirect techniques including high resolution-mass spectrometry and impedance spectroscopy. Analytical detection can be oriented in-line with the OoC or the sample can be removed from the model system for off-line data collection. For example, an off-line analysis could include a sample being removed from the OoC and being analyzed via high-performance liquid chromatography (HPLC) (Pemathilaka et al., 2019a) versus an in-line analysis such as impedance biosensors embedded within the OoC model (Schuller et al., 2020). Early OoC models were limited with most analytical methods taking place off-line, which does not provide the rapid and continuous feedback loop necessary to monitor organ physiology. OoC models have advanced to include analytical instrumentation that can detect metabolic processes, cytokine biomarkers, inflammation, and membrane degradation within the OoC device. With analytical techniques, the identification and characterization of analytes within OoC models uncovers minute details that are beneficial for overarching connections experienced *in vivo*.

In this review, we describe fetal membrane and placental biology and provide background on human embryogenesis and the supportive structure of the reproductive organs. We explore OoC history, recent fetal membrane on-a-chip, placenta on-a-chip, reproductive tract on-a-chip, and *in vitro* fertilization on-a-chip advancements. We examine immunology and mechanistic experiments, and future OoC directions as the research expands to greater applications including PTB treatments, drug development, and *in vitro* fertilization (IVF) studies.

## 2 Human embryogenesis & fetal membrane biology

Human embryogenesis is an intricate process that begins with an egg and sperm and ends with a fetus. The process initiates with the fertilization of an oocyte with sperm to form a double zygote, which divides and replicates (Gerri et al., 2020). The increased number of cells, termed blastomeres, adhere together to form a blastocyst while cavitation, or the formation of the fluid-filled amniotic cavity, occurs (Gerri et al., 2020). By 7–10 days post-fertilization, the blastocyst develops into an embryo to travel through the Fallopian tubes towards the uterus and implant into the endometrium (Gerri et al., 2020).

**FIGURE 1:**
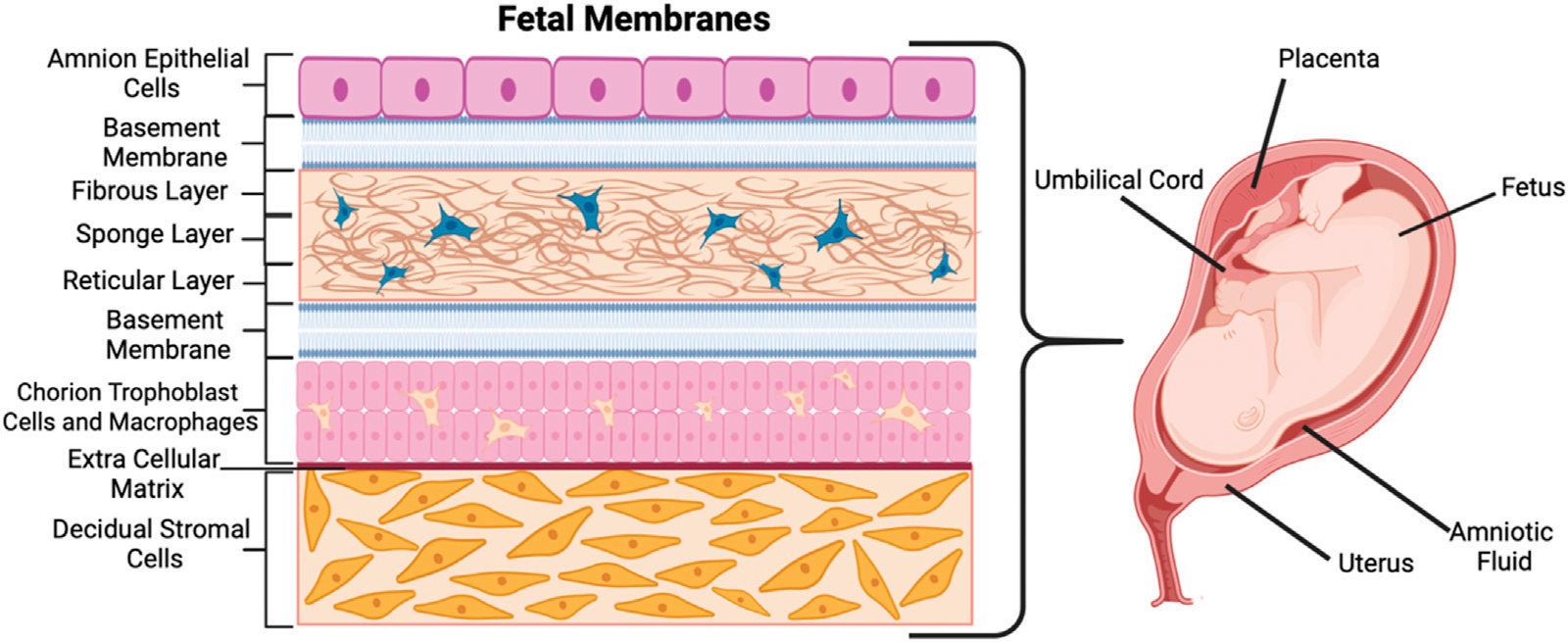
Schematic of the fetal membranes and their corresponding architectures and cell types. Created with BioRender.com.

The blastocyst compartments include (1) trophoblast cells and (2) the inner cell mass (Rossant and Tam, 2022). The inner cell mass divides into three germ layers during a reorganization process known as gastrulation (Rossant and Tam, 2022). The three germ layers, the endoderm, ectoderm, and mesoderm form the lining of the internal organs, brain/nervous systems/external tissues, and the muscles/circulatory system/skeletal system, respectively (Rossant and Tam, 2022). The first compartment—the trophoblast cells—go on to form the fetal membranes and the placenta (Figure 1).

Often called the fetal membrane (singular), the trophoblast cells divide into the four extra-embryonic membranes—the amnion and chorion membranes, the yolk sac, and the umbilical cord (Carlson, 2023; Herrick and Bordoni, 2023; Richardson and Menon, 2022). The first, the amniotic membrane, is composed of amnion epithelial cells (AECs) and mesenchymal cells (AMCs) which form the sac that surrounds the embryo (Carlson, 2023). The amnion layer (shown in Figure 1 as the top purple-colored layer) lines the inner surface of the amniotic sac that holds the fetus, amniotic fluid, and the connecting stalk umbilical cord (Carlson, 2023; Lei et al., 2017). The amnion acts as an extra-embryonic membrane that protects the fetus from maternal immune responses (Shahbazi, 2020). The chorion is the second membrane and the outer layer from which the placenta develops (Figure 1) (Lei et al., 2017). It is composed of the chorion trophoblasts and chorion mesenchymal cells (CMCs) and is a metabolically active tissue that functions as a barrier for mineral transport (Lei et al., 2017). Between the amnion and chorion are various protective layers including the fibrous layer, sponge layer, reticular layer, and basement membranes (middle, blue and cream-colored layers in Figure 1). A collagen-rich extracellular matrix (ECM) made up of fibrous proteins connects the amnion and chorion layers to the decidual stromal layer (bottom, orange-colored layer in Figure 1) (Menon and Richardson, 2017). The third membrane, the yolk sac, develops simultaneously with the amnion and forms outside the embryo (Hafez, 2017). The fourth membrane also forming outside the embryo, the allantois, is a sac-like membrane that takes part in the development of the umbilical cord (Bazer and Johnson, 2018). The chorion layer (last layer before the ECM in Figure 1) continues to grow to form the placenta, which has its own individual and unique biology.

## 3 Placental biology

The placenta develops from the placental cytotrophoblast to form the syncytium and cytotrophoblast subsets that include villous, extravillous, and syncytiotrophoblasts (Carlson, 2023; Herrick and Bordoni, 2023). The cytotrophoblasts differentiate into the chorion trophoblasts, which adhere to the amnion membrane (Carlson, 2023; Herrick and Bordoni, 2023). The various villi continue to grow and expand forming the placental shell (Herrick and Bordoni, 2023). As progesterone hormone increases, the maternal decidua basalis cells develop and protect the uterus from the overgrowth of the syncytiotrophoblasts (Carlson, 2023; Yin et al., 2024). The amniotic sac and chorionic sacs merge to form the amniochorion, which is the membrane that ruptures during labor, and the amniochorion fuses to the maternal decidua parietalis (Carlson, 2023; Yin et al., 2024).

The decidua is a temporary organ that is shed with the placenta after birth. It comes from the endometrium, the membrane lining of the uterus, and has three sections—the decidua capsularis, decidua parietalis, and decidua basalis (Carlson, 2023; Yin et al., 2024). The decidua basalis anchors the villous placental trophoblasts, forming a fetal-maternal interface, or a junction at which transport moves from the mother to the fetus (Balasundaram and Farhana, 2023). This interface is a target of mechanistic transport and biological pathway investigations of potentially toxic metabolites that could activate PPROM and PTB.

The placenta’s primary function is to supply the growing fetus with hormones, oxygen, nutrients, and waste removal via transport and metabolic pathways (Gude et al., 2004; Cindrova-Davies and Sferruzzi-Perri, 2022). Its mechanisms are known to be a complex combination of dialogue between protein expression, transmembrane gradients, and placental blood flow (Burton and Fowden, 2015). When these pathways are disrupted and the pregnancy is complicated by placental dysfunction and nutrient depletion, the fetus could be negatively impacted (Zhou et al., 2023). It is known that the mechanisms of placental exchange include diffusion, transporter-mediated mechanisms, and endocytosis/exocytosis, but elucidating exactly how these mechanisms take place and their specific interactions with the fetal membranes are still future goals (Burton et al., 2016).

**FIGURE 2:**
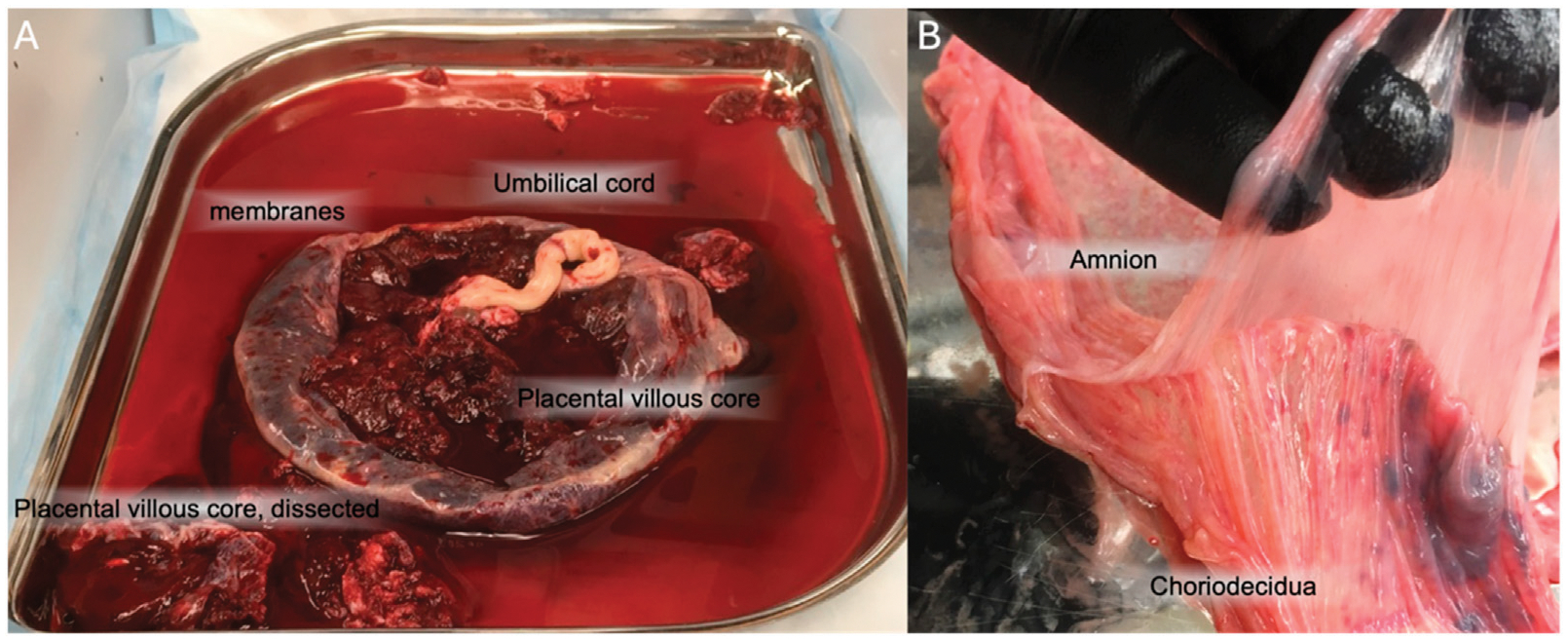
Gross anatomy of the placenta and fetal membranes. **(A)** the placental disc is largely comprised of the placental villous core, primarily CTBs. The fetal membranes come off the placenta. The umbilical cord is often found in the center of the disc. **(B)** The fetal membranes, removed from the placenta, can be manually separated into amnion and choriodecidua.

Placental and fetal membrane organs are non-redundant. While interconnected, the placenta and the fetal membranes are separate organs (Figure 2), with distinct cell types that serve distinct functions-namely, nutrient and oxygen exchange, and mechanical and physical barrier purposes, respectively (Kay et al., 2011). While the placenta has cell types termed cytotrophoblasts (CTBs), the fetal membranes have chorion trophoblast cells (CTCs) that have differentiated from CTBs. The placental CTB subsets include villous, extravillous, and syncytiotrophoblasts; these have distinct properties relative to the CTCs of the fetal membranes (Bischof and Irminger-Finger, 2005). Additionally, the amnion and mesenchymal layers of the fetal membranes are unique and highly immunomodulatory—where the chorion is known for its immune regulatory roles, the amnion has been shown to play a larger role in tensile strength and barrier function (Insausti et al., 2014)—making independent study of the placenta and fetal membranes essential.

PTB and PPROM is often stimulated by factors such as mother’s age, weight, prenatal care, smoking, alcohol, drug abuse, hypertension or acute chorioamnionitis (CAM) (Halimiasl et al., 2017). CAM is inflammation of the fetal membranes, which can be from sterile inflammation of unknown causes or infectious stimuli such as bacteria (Eastman et al., 2022). Specifically, the infection and inflammation can occur at the interface between the fetal and maternal tissues. Approximately 10% of all laboring women experience CAM and are treated with antibiotics, but still risk significant complications to the mother and fetus (Hastings-Tolsma et al., 2013). CAM is associated with an array of adverse pregnancy outcomes, including perinatal death, pneumonia, neonatal septic shock, and intraventricular maternal hemorrhage (Czikk et al., 2011; Jain et al., 2022). Once CAM is detected, treatment typically includes antibiotics such as ampicillin and gentamicin to address potential infectious sources, but all complications are not likely completely resolved with antibiotic administration (Conde-Agudelo et al., 2020). Many studies have worked to identify biomarkers such as cytokines, chemokines, and pathogen-or damage-associated molecular patterns that may signal *in-utero* inflammation or CAM, allowing researchers to better develop timely medical intervention (Redline, 2012). The current treatments remain insufficient, hence the demand for organ-on-chip technologies (OoC).

## 4 OoC background

External platforms that mimic fetal organs, such as reproductive OoC models, are well poised to be the frontier of reproductive research. Through reproductive research platforms, placental transport studies could provide deeper insight into drug transport mechanisms (Ma et al., 2021). For example, in the 1962 thalidomide tragedy, the prescription drug thalidomide was found to permeate through the placental barrier and severely alter fetal development causing significant limb malformations (Annas and Sherman, 1999; Arumugasaamy et al., 2020). Prior to this event, it was popularly believed that the human fetus was completely protected from maternal drug exposures (Annas and Sherman, 1999; Dally, 1998). Reproductive organs can be precisely mimicked on a 3D device allowing for drug monitoring, transport mechanism analysis, and biomarker detection (Richardson et al., 2020a). Studying drug interactions and pathways with reproductive organs unique to the duration of pregnancy, is highly crucial, yet pregnant women are often excluded from clinical studies, understandably so, as the life of the mother and fetus could be put at risk. To address this, other modes of reproductive research include ultrasounds, two-dimensional cell cultures, and animal models.

Ultrasound technology and 2D culture were revolutionizing techniques that altered the progression of research at their time. Ultrasound technology is used as a low-risk technique that allows for image investigations of the fetal-maternal functions, but it fails to show cellular or mechanistic functions. To examine cell functions directly, 2D cell culture—which is the growth of a monolayer of cells on the surface of a culture flask or petri dish—provided a beneficial foundation for basic concepts. 2D cell culture became widely popular in the 1950s with the establishment of the immortal HeLa cell line, leading to the advanced understanding of disease pathways, drug designs, and toxicology (Singh et al., 2022). As with all new designs and discoveries, researchers began to realize the limitations that come with 2D cell culture and the need to move to more biologically precise 3D models (Richardson et al., 2020a; Duval et al., 2017; Jensen and Teng, 2020; Sun et al., 2021). Lastly, the results of 2D cell experiments often contradict those obtained from *in vivo* responses and animal models (Bédard et al., 2020).

Animal experiments involve ethically using an animal model, typically mice or rodents, to conduct scientific research. Animal models do display complex biochemical interactions and serve as a useful tool for scientific advancements—such as the development of the polio vaccine with monkeys (Robinson et al., 2019) and contraceptives with rabbits (Castle et al., 1998). However, the cost of animal model research is high and ethically requires special handling (Barré-Sinoussi and Montagutelli, 2015; Van Norman, 2019). Regarding reproductive research, human embryogenesis and the human fetal microenvironment differs largely from that of other mammalian models (Gerri et al., 2020). Reproductive research has heavily used pregnant sheep to model maternal-fetal interactions, but the model is still unable to truly recapitulate human pregnancy (Barry and Anthony, 2008). Another common model for human placental research is mice as they are small, have large litter sizes, and have a short gestation time (Carter, 2020; Maltepe et al., 2010). However, much of mice organ development takes place post-birth, making the mouse model an inferior model for obstetrical syndromes arising in the third trimester (Carter, 2020; Maltepe et al., 2010). Additionally, maternal blood in the human placenta perfuses the intervillous space, whereas in rodents, and many other mammals, the exchange is between fetal and maternal capillaries (Carter, 2020; Maltepe et al., 2010). Human gestation and birth are unique and 3D organotypic models provide the canvas to explore complex recreation.

3D cell culture grew in popularity in the late twentieth century. The first OoC platform was a lung-on-a-chip model developed in 2010 by Donald E. Ingber and co-workers (Sood et al., 2023). Since then laboratories have worked to develop brain (Herreros et al., 2022; Amirifar et al., 2022), liver (Liu et al., 2022; Dalsbecker et al., 2022), heart (Jastrzebska et al., 2016; Abulaiti et al., 2020), stomach (Lee et al., 2018; Ferreira et al., 2023), kidney (Wang et al., 2022; Ashammakhi et al., 2018), and bladder (Sharma et al., 2021; Galateanu et al., 2022), among many other OoC devices (Sosa-Hernández et al., 2018; Zarrintaj et al., 2022). The field has rapidly advanced with new innovative designs to incorporate pluripotent stem cells and even multiple organs in one model, which could be valuable in preclinical trials (Picollet-D’hahan et al., 2021; Ingber, 2022; Safarzadeh et al., 2024a). Out of the various organ models, placental and fetal membrane models, specifically, are far less frequent and thus highly beneficial to reproductive investigations.

## 5 OoC models

In this review, we describe 20 reproductive OoC platforms published after 2017, as previously written reviews have investigated preceding devices (Richardson et al., 2020a; Gnecco et al., 2018; Elzinga et al., 2023). The models, organized in Table 1, are categorized by fetal membrane on-a-chips (five models), placenta on-a-chips (nine models), and multiple reproductive OoCs (six models). For each model publication, the experimental objectives, chip fabrication techniques, analytical methods, and significant outcomes are explored. Recent model developments have greatly impacted the fields of bioengineering, microfabrication, and obstetrics and gynecology.

## 6 Fetal membrane models

The fetal membrane on-a-chips (FMOC) are platforms modeled after OoC technology that incorporates various combinations of the amnion and choriodecidual tissues onto a substrate of choice. Different conditions, stressors, and toxins can be introduced into the platform, and the transport mechanisms and immune responses can be examined through various analytical instrumentation techniques. The goal is that FMOC researchers can replicate enough aspects of the reproductive system to advance current mechanistic understandings.

**FIGURE 3:**
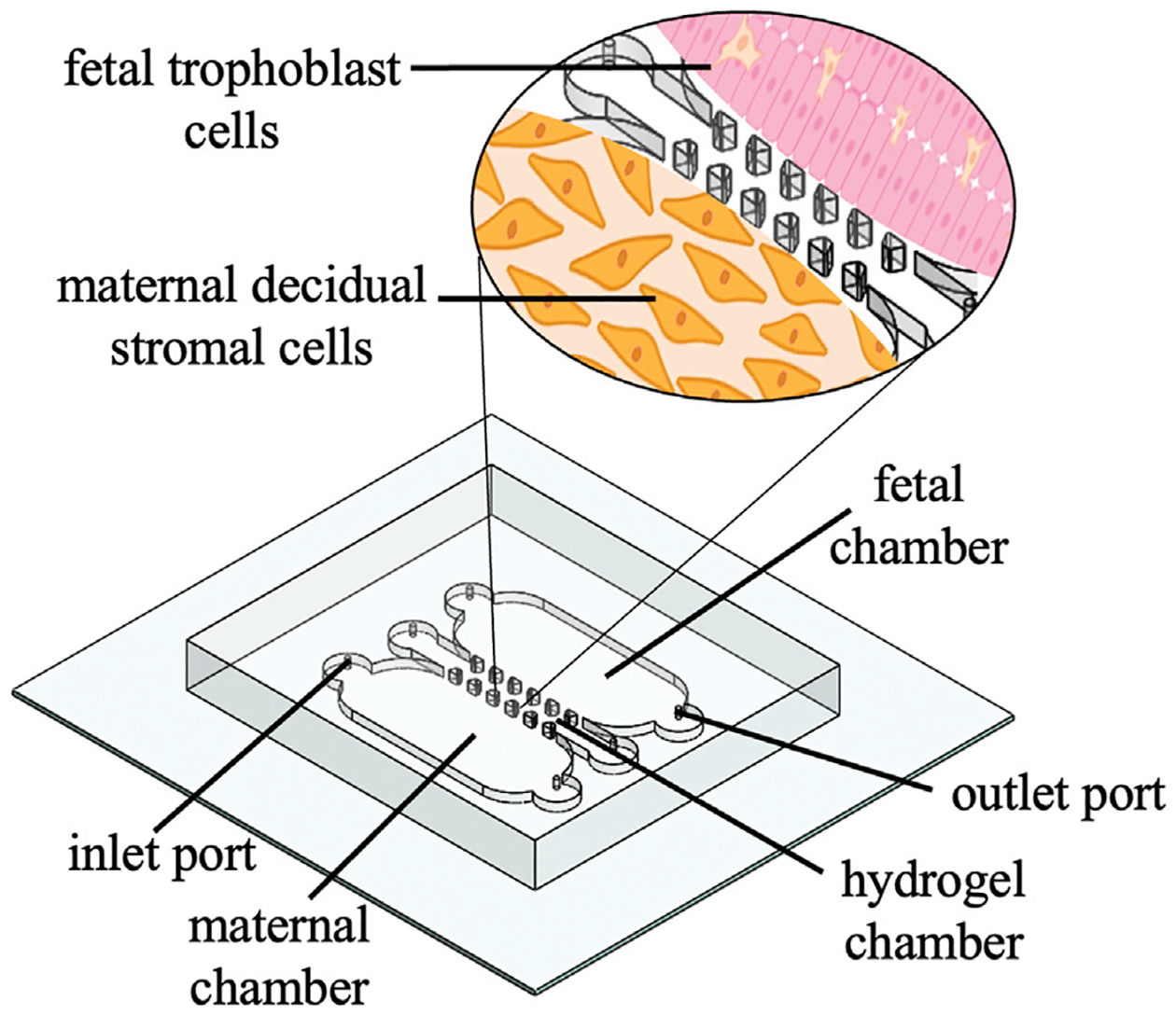
Schematic of a 3D printed PDMS butterfly model. Schematic shows inlet and outlet ports, a hydrogel chamber that mimics the ECM, and a zoom in of the fetal chamber (pink trophoblast cells) and maternal chamber (orange decidual stromal cells).

Previous reviews by Gnecco et al. and Eastman et al. briefly highlight FMOC methods and an instrumented fetal membrane on-a-chip (IFMOC) (Eastman et al., 2022; Gnecco et al., 2018). This design allows for the integration of real-time electrical sensors to assess 2 cell’s individual contributions to fetal membrane function. The model was utilized for the investigation of Group B *Streptococcus* infected decidual stromal cells and macrophage activation (Eastman et al., 2022). This device consisted of a flat, planar model with a collagen coated membrane to mimic the ECM and resulted in several advantages, including cell visibility, imaging capacity, and cell-to-cell crosstalk (Eastman et al., 2022). Our laboratory is currently experimenting with a butterfly orientation of this model to electrochemically detect biomarkers which may signal for PPROM (Figure 3).

The PDMS butterfly model has been fabricated based upon 3D printing techniques previously described by O’Grady and co-workers (O’Grady et al., 2021). The device contains two horizontal microfluidic chambers, one for decidual stromal cells and one for trophoblast cells, divided by a permeable hydrogel chamber, to mimic the chorion membrane at its fetal-maternal interface. The foundation of at least two microfluidic chambers divided by a permeable membrane is notably the most common design within this review.

Richardson, Menon, and coworkers have significantly contributed to the field with numerous studies incorporating the amnion fetal membrane, specifically the development of the amnion membrane organ-on-chip (AM-OOC) model. The AM-OOC was composed of two chambers connected by type IV collagen-coated microchannels to examine the interactive and transition properties of primary human amnion epithelial cells (AECs) and amnion mesenchymal cells (AMCs) (Richardson et al., 2019). The device was assembled with PDMS oxygen-plasma bonded onto a glass substrate, and the inner microfluidic chambers were coated with Matrigel to mimic the amnion basement membrane *in vivo*. Fluidic isolation between the two microfluidic chambers was shown via a fluorescent dye perfusion assay. The authors subjected the model to oxidative stress by exposure to cigarette smoke extract exposure and/or N-acetyl-L-cysteine. It was discovered that oxidative stress in both microfluidic chambers promoted an inflammatory response and prevented migration (Richardson et al., 2019). The model design was limited by having only two microfluidic chambers, but advanced previous models with the incorporation of an ECM. The AM-OOC model enabled experimental manipulation of multiple cell types and provided a foundation for a further mechanistic understandings of cell behavior during pregnancy (Richardson et al., 2019).

In 2020 Richardson et al. then utilized cigarette smoke extract and dioxin to compare traditional transwell culture systems and a repurposed two-chamber fetal membrane organ-on-chip device previously fabricated by Gnecco et al (Gnecco et al., 2018). The PDMS device, termed the FM-OO-C, consisted of a porous membrane oxygen-plasma bonded over a top PDMS layer, followed by plasma-bonding of a second layer, orthogonally to the top PDMS layer (Richardson et al., 2020c). Primary amnion epithelial cells (AECs) and decidual stromal cells were co-cultured within the platform. By analysis of perfusion assays for cigarette smoke extract and dioxin, the authors determined that the FM-OO-C model had more membrane permeability than transwell systems (Richardson et al., 2020c). In the FM-OO-C model, treatments forced changes between cellular layers and improved signal propagation, suggesting sensitive cell-to-cell interactions and crosstalk, in comparison to traditional transwell platforms (Richardson et al., 2020c). This investigation was advantageous, but the FM-OO-C model lacked the fetal-maternal interface—a key point of interest. The authors concluded that the comparative analysis suggested the FM-OO-C platform is preferable for fetal membrane studies and advanced the model to include the fetal-maternal interface.

**FIGURE 4:**
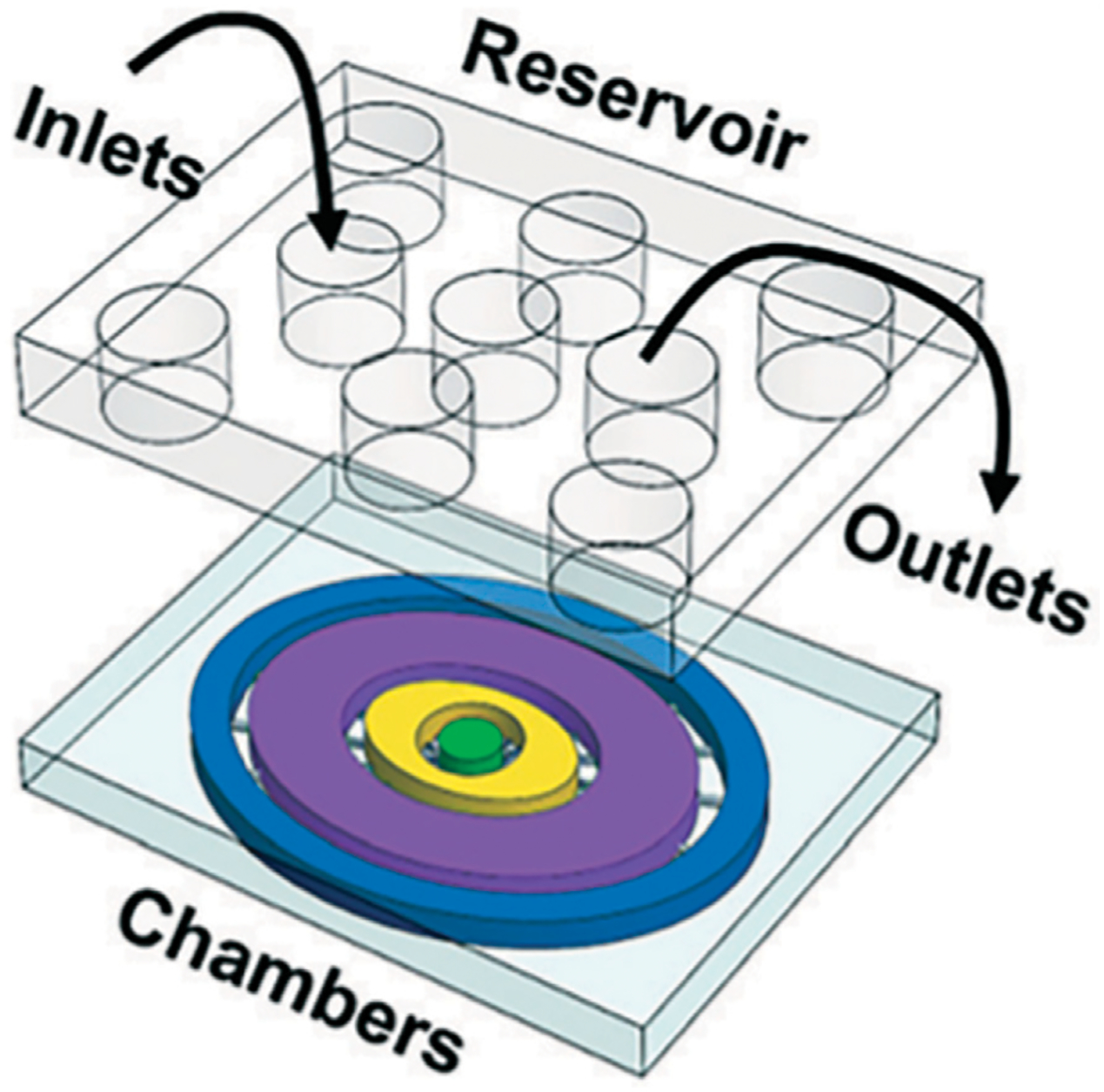
Schematic of Richardson and coworkers’ FMi-OOC model. The platform schematic shows each cell culture chamber having a different color for easy visualization. The choriodecidua interface (chorion-yellow; decidua-green) and the amniochorionic interface (amnion AMC-purple; amnion AEC-blue; chorion-yellow) chambers are connected by an array of 24 microchannels. An on-chip media reservoir for media diffusion control was aligned on top of the cell loading inlets and outlets. Figure D adapted from (Richardson et al., 2020b) with permissions from Lab on a Chip.

Next, Richardson et al., in 2020 published an updated platform modeling ascending infection of E. *coli* from maternal to fetal tissue. In the model, lipopolysaccharide (LPS) was used to model E. *coli* entering their fetal-maternal interface organ-on-chip (FMi-OOC) model (Figure 4) (Richardson et al., 2020b). The authors incorporated primary cells from the decidua, chorion, amnion mesenchyme (AMC), amnion epithelium (AEC), and the collagen rich matrix from full-term patients into a four-chamber co-culture model (Richardson et al., 2020b).

The fabrication materials were similar to previous developments, but each cell layer or chamber was interconnected through an array of 24 microchannels to provide a series of microbiological functions (Richardson et al., 2020b). Additionally, the platform included multiple reservoirs to supply the platform’s various cell chambers with cell culture media, but it did not include continuous media perfusion to incorporate biomechanical stressors such as dynamic shear stress (Richardson et al., 2020b). The authors fluorescently labeled LPS and imaged the fluorescent intensity as the LPS traveled through the chambers. LPS did pass through all the chambers, indicating that LPS can travel through the decidua, chorion, amnion mesenchyme and into the fetal amnion within 72 h (Richardson et al., 2020b). Imaging with immunocytochemical staining for LPS, NK-kB, cytokeratin-18, and vimentin, cell viability and matrix collagen staining showed detailed images of this infiltration. Additionally, pro-inflammatory cytokine biomarkers such as IL-6 and GM-CSF were detected throughout the cell layers at various time points, indicating the authors were able to successfully model ascending infection in the FMi-OOC device (Richardson et al., 2020b).

Advancing this study was a 2021 publication by Radnaa et al. incorporating the recently constructed FMi-OOC to investigate fetal-maternal signaling. The work focused on fetal-maternal signals that initiate parturition with the protein HMGB1, which is associated with PTB (Radnaa et al., 2021). The authors hypothesized that senescent amnion cells release HMGB1, a biomarker capable of increasing fetal-maternal interface inflammation leading to higher possibilities of PTB (Radnaa et al., 2021). The experiment included testing the migration of exosomal HMGB1, referred to as eHMGB1, through the four-chamber co-culture FMi-OOC model (Radnaa et al., 2021). The author’s hypothesis was confirmed via cytokine immunoassays that eHMGB1 traveled from the fetal cells to the maternal decidua increasing inflammation (Radnaa et al., 2021). Furthermore, the study was confirmed in a mouse model that showed the intraamniotic injection of eHMGB1 into pregnant mice leads to PTB (Radnaa et al., 2021). Following this study, the group examined the efficacy of the model in a toxicological investigation.

In 2022, Kim et al. published on the efficacy of the four-chamber FMi-OOC system to study the response of an environmental toxin cadmium on the fetal membranes (Kim et al., 2022). This study uniquely focused on an environmental toxin, an innovative approach in FMi-OOC studies. Cadmium distribution was tracked via ICR-mass spectrometry and the degree of cell death was quantified using apoptotic/necrotic markers (Kim et al., 2022). Bright field microscopy recorded cell morphology and multiplex cytokine assays detected inflammatory indicators (Kim et al., 2022). This model was limited to examining a one-time cadmium exposure rather than multiple long-term exposures, but it has the potential to be advanced into a dynamic media perfused platform. The author’s found that maternal cadmium exposure induced decidual apoptosis and inflammation, but the same was not always observed in fetal exposures (Kim et al., 2022). The chorion barrier did not allow for the propagation of cadmium within the FMi-OOC platform, therefore amniochorion cell death did not occur (Kim et al., 2022). This finding was not unexpected due to the fundamental purpose of the chorion barrier, but the results do prove the efficacy of the FMi-OOC model (Kim et al., 2022).

Ganguly et al., in 2021 and Richardson et al., in 2022 utilized the FMi-OOC model again to investigate organic anion transporting polypeptide 2B1 (OATP2B1) (Ganguly et al., 2021) and drug transport across both fetal-maternal interfaces (Richardson et al., 2022). These works studied the propagation of rosuvastatin with without oxidative stress by cigarette smoke extract, but were limited by omitting dynamic media perfusion. Both investigations demonstrated that the FMi-OOC platform can be used in clinical trials to understand the FM-decidua parietalis pathway, which helps improve drug delivery testing and design, improving PTB related treatments (Ganguly et al., 2021; Richardson et al., 2022). The recent work of the Menon laboratories has largely advanced the reproductive OoC field making significant advancements in PTB research.

Studies of the fetal-maternal interface including the amniochorion and decidua are new and important areas of research. It is arguably one of the most vital biological pathways and medical intervention methods for this region are scarce. An area of study more commonly investigated for drug development is the human placenta and the mechanisms within its fetal-maternal interface as mimicked on a placenta OoC.

## 7 Placenta on-a-chip models

As with the FMOC, various groups have created placenta on-a-chip platforms with different membrane materials to mimic various aspects of the placenta (Pemathilaka et al., 2019b; Cherubini et al., 2021; Cho et al., 2021). Previously written reviews (Gnecco et al., 2018; Elzinga et al., 2023; Pemathilaka et al., 2019c) have focused on groundbreaking placental models (Lee et al., 2016) which set the foundation for modern models. The placenta on-a-chip platforms reviewed in this publication showed significant advancements in robust model designs, barrier transport analyses, and the incorporation of trophoblast stem cells, largely furthering the field of reproductive OoC research.

Mandt et al., in 2018 created a custom placenta membrane barrier for a 3D placenta on-a-chip model using 3D printing techniques. The chip was created with poly-(ethylene glycol)-dimethacrylate, glass plates, and PDMS spacers. The barrier was fabricated using high resolution two-photon polymerization (2PP) and cell biocompatibility and cellular barrier response was investigated. Within this design, 2PP was advantageous for creating micrometer precision barrier structures to precisely mimic the placental barrier microenvironment (Mandt et al., 2024). Each side of a GelMOD barrier contained isolated cell culture compartments, one for human umbilical vein endothelial cells (HUVEC) and one for BeWo (materal) cells (Mandt et al., 2024). The cellular response, composition, resolution, and material stability was tested. With respect to membrane permeability, the authors found that smaller molecules around the size of glucose were able to diffuse through the barrier, but those of high molecular weights did not (Mandt et al., 2024). Additionally, viable cell count was higher when the placental barrier was coated with fibronectin than when not (Mandt et al., 2024). This experimental design proved advantageous for cell biocompatibility over designs that incorporate inorganic polymers to resemble the ECM and showed the importance of protein-coated microfluidic chambers (Mandt et al., 2024).

In 2019, Pemathilaka et al. used a placenta-on-a-chip model to investigate caffeine transport across the placental barrier. Caffeine concentration was quantified on the fetal side of the placental barrier after being introduced on the maternal side via high-performance liquid chromatography-tandem mass spectrometry (HPLC-MS-MS) (Pemathilaka et al., 2019a). The device fabrication included two PDMS layers and a polyester track etched membrane coated with entactin-collagen IV-laminin solution as the placental barrier. Then, this device was seeded with HUVECs (fetal) and BeWo (maternal) cells and the barrier permeability was examined via fluorescence intensity analysis. Caffeine was introduced from the maternal side and samples from both sides were collected every 30 min for HPLC-MS-MS analysis and the rate of caffeine transfer was calculated. The fetal caffeine concentration increased until it reached a steady state after five hours (Pemathilaka et al., 2019a). This study serves as a model for transport analysis, with dynamic perfusion, across the fetal-maternal interface and proved the ability of the OoC model to mimic transport as seen *in vivo*.

Another transport study was conducted in 2020 by Mosavati and coworkers investigating glucose diffusion across an experimental placenta-on-a-chip membrane and a numerical 3D model (Mosavati et al., 2020). The experimental model was fabricated with trophoblast cells and HUVECs cultured on opposite sides of a polycarbonate membrane (basement membrane substitute) that was sandwiched between two PDMS microfluidic channels (Mosavati et al., 2020). The authors studied the effects of flow rate and membrane porosity on the rate of glucose transfer and diffusion. Under the conditions tested, the rate of glucose diffusion increased with membrane porosity and decreased with flow rate (Mosavati et al., 2020). The author’s experimental monitoring of glucose transfer rates across the membrane was slightly less than that of COMSOL 5.2 simulated data, but the small deviation still showed good agreement between experimental and numerical results (Mosavati et al., 2020). This was an important study to help understand the physics of flow and transport in addition to validating numerical and experimental models.

In 2022 this same group examined nutrient exchange and glucose transport during infection using a placenta-on-a-chip model (Mosavati et al., 2022). The authors describe the fetal-maternal interface model as a commercial three-lane OrganoPlate microdevice. The design included BeWo and HUVEC cell culture chambers separated by ECM gel, with each channel connected to 2 cell media reservoirs (Mosavati et al., 2022). The device was placed onto a rocking platform to create a bidirectional flow under shear stress for the uniform lining of cells inside the channels (Mosavati et al., 2022). A glucose gradient was introduced via the culture medium and the glucose transport across the ECM was measured in the presence and absence of chondroitin sulfate A (CSA)-binding *P. falciparum* infected erythrocytes, an event that occurs in placental malaria (Mosavati et al., 2022). Many researchers typically fabricate their own OoC platforms, however this study is an example of a commercial chip platform. This commercial chip platform may be useful for developing placental malaria treatments (Mosavati et al., 2022). Perhaps just as importantly, this investigation showed how the chip could be altered to fit the conditions of a specific study, substantially decreasing experimental times and yielding reproducible and comparable results.

In 2023 Ghorbanpour et al. also used a commercial 3D placenta-on-a-chip model, this time to investigate immune responses during preeclampsia (Ghorbanpour et al., 2023). The model included HUVECs and a first trimester trophoblast cell line (ACH-3P) to investigate the signaling and mechanisms of FKBPL and Gal-3, which are inflammatory proteins implicated in preeclampsia (Ghorbanpour et al., 2023). The cyclic olefin polymer platform, purchased from AIM Biotech (Singapore), contained three microfluidic chambers including one ECM central channel, with all chambers under interstitial flow. For analysis, the authors conducted immunofluorescence staining, laser scanning confocal microscopy, western blotting, and ELISAs. It was determined that FKBPL and Gal-3 are present in increased concentrations in preeclampsia positive samples (Ghorbanpour et al., 2023). Additionally, protein expression patterns were impacted by cellular interactions, and inflammation of both proteins was associated with impaired vascular network formation, both contributing to preclampsia (Ghorbanpour et al., 2023). This proof-of-principle study was useful for demonstrating trophoblast invasion, biomarker discovery, and drug analysis. Finally, this work showed how a commercial 3D model could be used as a low-cost alternative to mimic the human placenta.

Another commercial 3D microfluidic chip comparative analysis was conducted by Pu et al., in 2021 to evaluate fetal trophoblast invasion with various matrix materials (Pu et al., 2021). The device consisted of a commercially fabricated PDMS microfluidic chip under shear stress with media flow, seeded with HTR8/SVneo, HUVECs, and various membrane matrices including gelatin, Matrigel, and fibronectin (Pu et al., 2021). The authors conducted permeability assays, matrix degradation assays, real-time quantitative PCR, transwell invasion assays, and flow cytometry. It was determined fibronectin had the highest cell attachment for both cell lines, followed by Matrigel and gelatin (Pu et al., 2021). The authors suggested that the high cell adhesion of fibronectin may be placental-specific, as fibronectin is secreted from placental cells and enables growth of both trophoblasts and endothelial cells (Pu et al., 2021). Other studies have developed OoC models utilizing Matrigel (Cho et al., 2021; Biglari et al., 2019; Utagawa et al., 2022) and gelatin (McCain et al., 2014; Pitingolo et al., 2019), but their chemical composition and limited flexibility likely make fibronectin an optimal matrix for replicating the ECM in this experiment. To fully optimize all aspects of the platform, some laboratories have developed devices which incorporate multiple matrix materials into the microfluidic device (Leung et al., 2022; Nahak et al., 2022; Shyam et al., 2023).

In 2022 Ko et al. used a gel-patterned system to analyze the changes in cell mobility with differences in oxygen concentration. While most devices primarily use PDMS for its low stiffness and oxygen control capabilities, the microfluidic chip in this experiment was fabricated with gelatin methacrylate (GelMA) to examine cell movement within a high stiffness material. The design consisted of microfluidic chambers stacked vertically with GelMA solution injected into the chamber for gel patterning (Ko et al., 2022). Despite this design being more limited for its complex fabrication, the authors successfully examined trophoblast migration through cell culture and controlled oxygen concentration though a hypoxic chamber. After quantitative RT-PCR, cell tracker staining, and fluorescence diffusion analysis, the authors determined that the mobility of trophoblast cells was upregulated, suggesting that a hypoxic environment in the endometrium contributed to an increase in cell mobility (Ko et al., 2022). Previous experiments conducted by Cho et al. similarly found that during long-term hypoxic conditions, cells excrete higher concentrations of MMP-9, which could promote degradation of the ECM (Cho et al., 2021). Both works investigate cellular behaviors during oxygen-stress conditions, uncovering details which many experiments fail to study.

**FIGURE 5:**
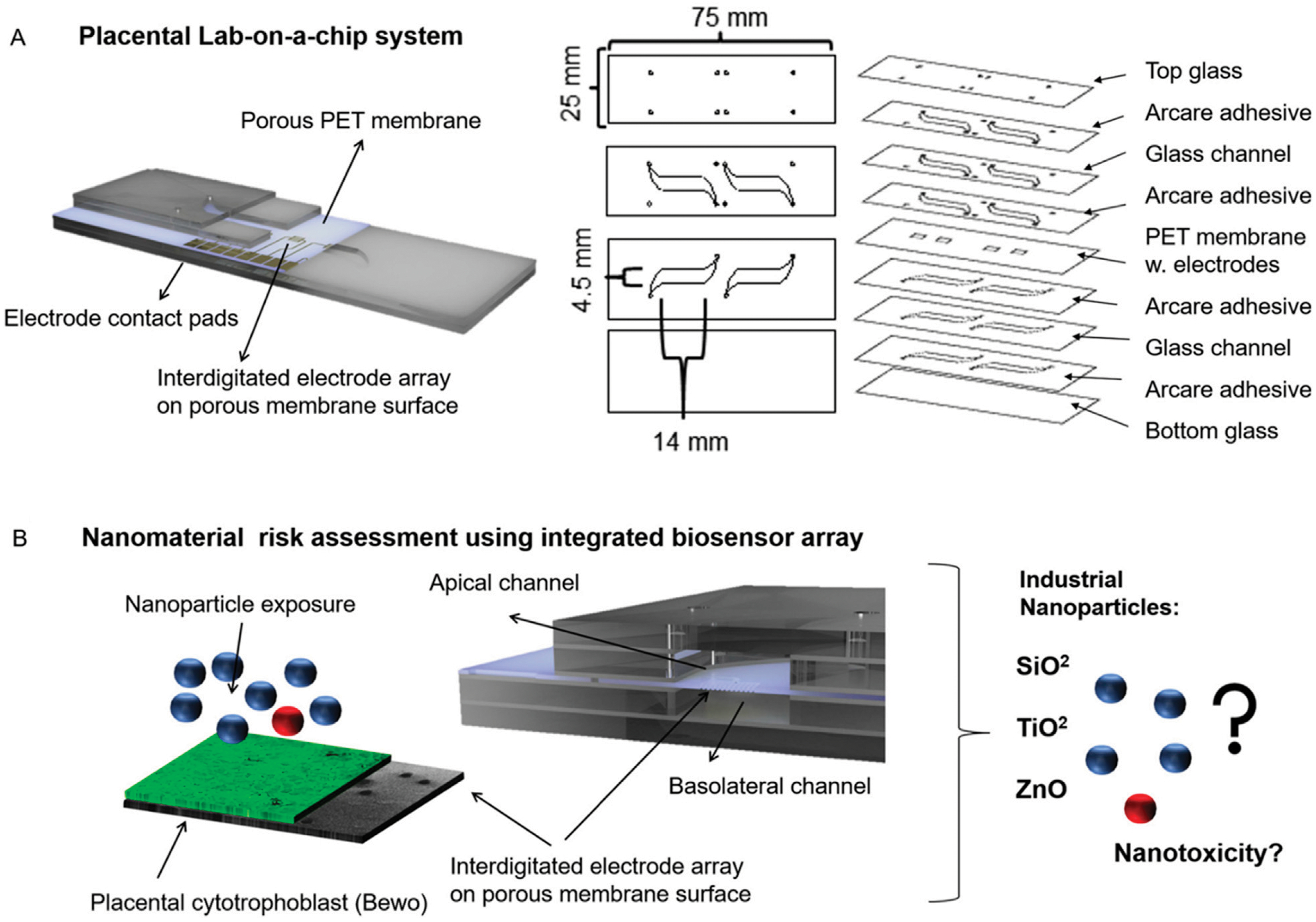
Schematic of Schuller and coworkers’ placental lab-on-a-chip with an interdigitated electrode impedance biosensor array fabricated on porous PET membranes **(A)** and the nanoparticle risk assessment with three industrial nanoparticles **(B)**. Figure adapted from (Schuller et al., 2020) with permissions from Sensors and Actuators B: Chemical.

In 2020, Schuller et al. conducted a nano-risk assessment of the placental barrier with the three most encountered nanoparticles (Schuller et al., 2020). The laboratory utilized a placenta-on-a-chip system (Figure 5) containing embedded membrane-bound impedance microsensor arrays capable of monitoring placental barrier transport and function during real-time nanomaterial exposure (Schuller et al., 2020). This placenta on-a-chip device contained an interdigitated impedance biosensor located on top of free-standing porous PET membranes and placental BeWo cells.

The nano-risk assessment was conducted using silicon dioxide, titanium dioxide, and zinc oxide, all which could potentially cause adverse effects in humans (Schuller et al., 2020). The performance of the electrochemical sensor was characterized using fluorescent dextran permeability assays, tetrapolar trans-epithelial electrical resistance (TEER) measurements, cyclic voltammetry, and impedance spectroscopy (Schuller et al., 2020). It was confirmed that the interdigitated device follows traditional, off chip trans-epithelial electrical resistance measurements and reactive oxygen species measurements for nano toxicological risk assessment (Schuller et al., 2020). An important aspect of electrochemical studies is the ability to conduct in-line analysis of analytes within the OoC platform. By monitoring conditions directly within the microfluidic, the user can obtain simultaneous results, giving a more precise view of the functions *in vivo*.

Recently in 2024, Hori et al. also utilized TEER to examine mother to fetus barrier function of syncyotiotrophoblast cells formed from trophoblast organoids using human trophoblast stem (TS) cells (Hori et al., 2024). Human trophoblast organoids that mimic the structure of placental villi are a novel 3D alternative to 3D perfusion on-chip models for investigating drug transport and drug development (Hori et al., 2024). The authors employed a 3-step differentiation treatment to successfully form spherical trophoblast organoids and develop a column-type barrier device (Hori et al., 2024). TEER measurements were performed on a co-culture barrier model with HUVECs and the models were validated using antipyrine, caffeine, and glyphosphate (Hori et al., 2024). Lermant et al. previously achieved similar developments of a placental barrier on-a-chip model with human-induced pluripotent stem cells (hiPSC)-derived trophoblasts (Lermant et al., 2023). In a perfused 3D OrganoPlate 3-lane device, the hiPSC-derived trophoblast arranged into a 3D structure expressing various placental transporters (Lermant et al., 2023). These results also demonstrated the use of stem cells in placental barrier modeling. Both experimental models proved to extend the utility of future placenta on-a-chip investigations.

The works described within utilized customized commercial chips and distinctive model designs to enhance placental barrier transport studies. To our knowledge, at the time of this review the placenta on-a-chip has yet to be used in a clinical trial that led to an approved FDA drug, but optimizing placenta on-a-chip fabrication conditions and transport studies pushes the field towards that desired outcome. Furthermore, while the most recent placenta OoC developments are highly promising, it is crucial to incorporate multi-reproductive organ models to precisely mimic cell crosstalk between multiple cell layers. Few studies have developed multi-reproductive organ models as that design must be highly intricate to recapitulate the full reproductive system. Laboratories which have developed multi-reproductive OoC platforms have greatly contributed to a largely understudied area.

## 8 Multiple reproductive OoC models

Human reproduction is an elaborate event in which specialized tissues and organs collaboratively, yet independently support fetal development. Advancing the understanding of this process has led to the design of new technologies, such as multi-reproductive organ models. Once impregnated, the amniotic sac, yolk sac, umbilical cord, and placenta develop with unique interconnected cellular crosstalk, which can be modeled in microfluidic platforms such as the reproductive tract on-a-chip.

**FIGURE 6:**
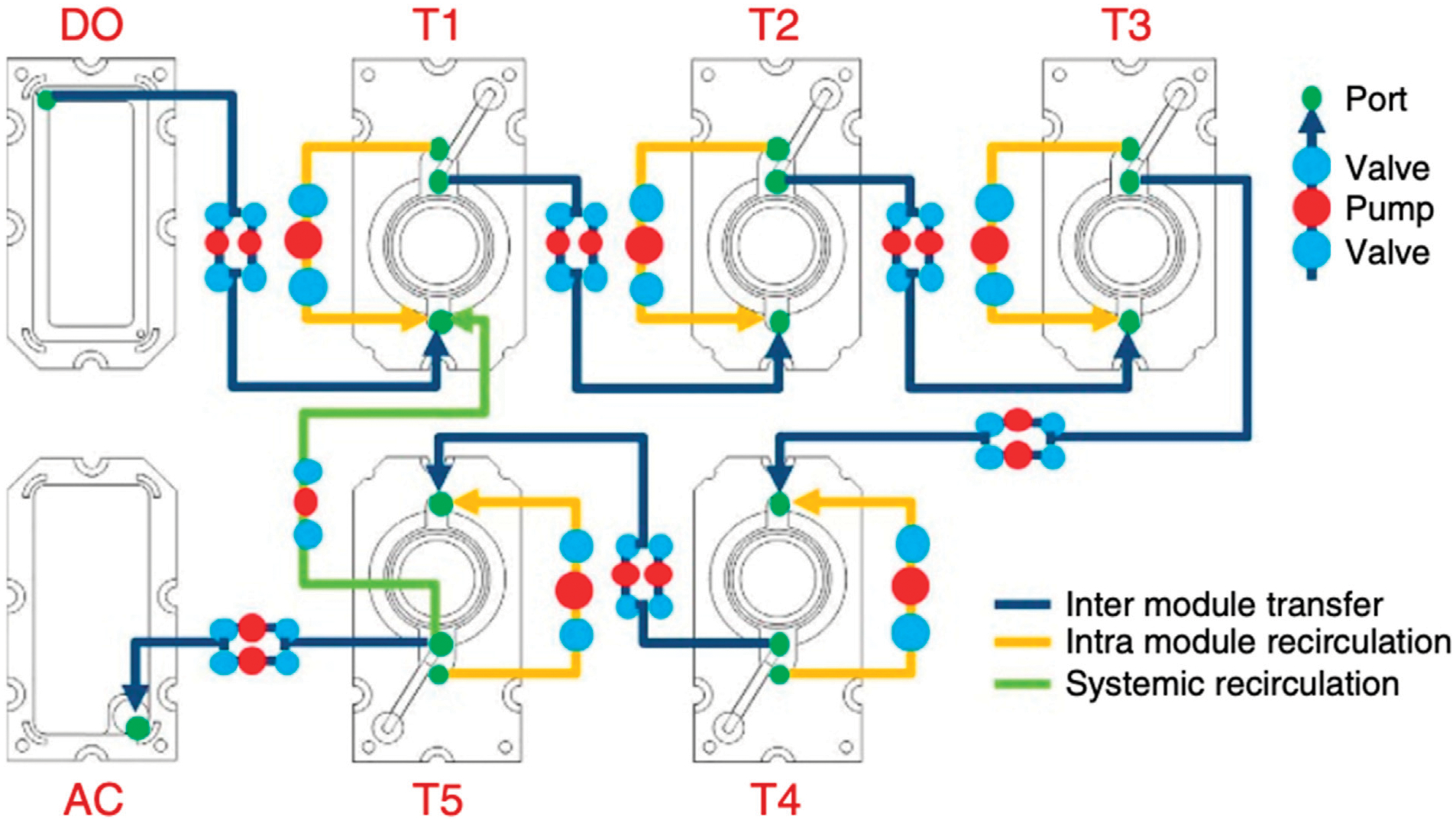
Schematic of Xiao and coworkers’ EVATAR quintet-MFP system showing tissue modules (T) one through five, an acceptor module (AC) and donor module (DO). Figure C adapted from (Xiao et al., 2017) with permissions from Springer Nature.

In 2017 Xiao et al. published on the development of their microfluidic platform, EVATAR, which stimulates the *in vivo* female reproductive tract modulating the ovary, uterus, cervix, fallopian tube, and liver (Xiao et al., 2017). The Woodruff laboratory is recognized as the first pioneer of an OoC model which fully recapitulates the entire 28-day menstrual cycle *in vitro*, an achievement termed EVATAR—named from the biblical “Eve” and a representative “avatar”. The EVATAR system allows for organ-to-organ hormone signaling with a sustained circulating flow between five-specific tissues secured on a fluidic plate that contains microfluidic channels (Figure 6) (Xiao et al., 2017). The full device consisted of a Quintet-microfluidic platform (MFP) with embedded electromagnetic actuation technology, plates, pumps, and valves to incorporate the various tissues into one interconnected system controlled with LabVIEW software. Each tissue, one through five, was designated to an individual microfluidic module connected to an acceptor module and donor module. Preliminary work included experiments in a Solo-MFP and a Duet-MFP, of one and two tissues, respectively, before incorporation into the Quintet-MFP. The Solo-MFP was used to examine follicle growth, 28-day hormone production, and subsequently, ovarian explant cultures in both MFPs. After the Solo-MFP and a Duet-MFP proved successful, the Quintet-MFP was used for five tissues.

The authors determined that the systems supported the investigation of cytokine expression, pregnancy-like hormone control, and liver metabolic studies (Xiao et al., 2017). The model proved successful and the first of its kind, largely advancing work in the field. Additional work of the Woodruff laboratory includes utilizing the EVATAR model as a platform for implanting human papilloma virus infected tissues to study cervical cancer (Reardon, 2017). A similar alternative to this work includes the advancement of previously designed two-chamber co-culture models to four-chamber models to mimic multiple reproductive layers.

Unlike any of the previously mentioned experiments, a study by Tantengco et al., in 2022 combines a vagina-cervix-decidua organ on-a-chip (VCD-OOC) and a four-chamber FMi-OOC model (Tantengco et al., 2022). The investigation was conducted to determine if exosomes from ectocervical epithelial cells contained *U. parvum* (*Ureaplasma parvum*), a commensal bacterium in the female genital tract, and whether this caused inflammation at the fetal-maternal interface (Tantengco et al., 2022). The VCD-OOC platform consisted of cells from the vagina, ectocervical, endocervical, transformation zone epithelia, cervical stroma, and the decidua in six interconnected microchannels (Tantengco et al., 2022). The VCD-OOC platform was fabricated very similarly to the FMi-OOC design with the five vagina-cervix epithelial-decidua chambers interconnected by an array of 24 microchannels, which are designed to mimic the basement membrane of the cervix (Tantengco et al., 2022). The cervical epithelial chambers were connected to a single large cervical stromal chamber by 72 microchannels (Tantengco et al., 2022). The supernatant from the decidua chamber of the VCD-OOC was added to the decidual chamber of the FMi-OOC platform after the vaginal chamber of the VCD-OOC was inoculated with exosomes produced by *U. parvum*-infected cells (Tantengco et al., 2022). The media from each of the four FMi-OOC microfluidic channels was examined via multiplex cytokine assays. The results indicated the increased presence of cytokines such as IL-6 and IL-8 (Tantengco et al., 2022). The exosomes initiated inflammation in the cervical and decidual cell layers, but not in the amnion mesenchymal, amnion epithelial, or chorion trophoblast cell layers of the fetus (Tantengco et al., 2022). Based on these results, the authors concluded the *U. parvum* infected exosome treatment was likely insufficient in initiating PTB (Tantengco et al., 2022). This model failed to incorporate immune cells and endocrinologic stimulation, but it still largely advanced the understanding of exosome’s functional effects on the fetal-maternal interface.

Recently, Menon and coworkers have integrated their models to form a multi-organ feto-maternal interface on-a-chip, termed the FMi-PLA-OOC (Safarzadeh et al., 2024b; Kammala et al., 2023). The model contained various microfluidic channels to allow for cell-to-cell crosstalk amongst seven different cell types. Pravastatin, a drug to reduce inflammation during pregnancy, transfer rate was examined at both feto-maternal interfaces and examined to previous models (Kammala et al., 2023). The FMi-PLA-OOC was also used to investigate the response of engineered extracellular vesicles containing interleukin-10 (eIL-10) on a lipopolysaccharide infected model (Safarzadeh et al., 2024b). The results showed that eIL-10 was effective at reducing inflammation and proved the FMi-PLA-OOC platform advantageous to animal models (Safarzadeh et al., 2024b). These works are novel advancements in multi-reproductive microfluidic modeling.

An early pioneer of organ on-a-chip modeling, Ingber, Mahajan, and coworkers, have developed a vagina on-a-chip to model vaginal microbiome-host interactions, such as that of anaerobic bacteria species found in bacterial vaginosis (BV) (Mahajan et al., 2022). Their microfluidic chip consisted of primary vaginal epithelium interfaced with stromal fibroblasts, with the addition of living microbes, in a two-channel PDMS device obtained from Emulate Inc (Mahajan et al., 2022). The authors introduced optimal *L. crispatus* and nonoptimal *Gardnerella vaginalis* and examined barrier integrity, lactate levels, pH, and cytokine excretion (Mahajan et al., 2022). In 2024, the laboratory modeled similar host-microbiome interactions in a human cervix on-a-chip model (Izadifar et al., 2024). The commercial Emulate Inc. chip was designed to replicate the human cervical mucosa lined by primary cervical epithelium interfaced with cervical stromal fibroblasts (Izadifar et al., 2024). As in the 2022 investigation (Mahajan et al., 2022), the same microbial communities were introduced, and immune responses, barrier function, cell viability, and mucus composition were determined comparable to previous *in vivo* observations (Izadifar et al., 2024). Both of these works demonstrated the use and advantages of preclinical on-chip models to accelerative future reproductive therapeutic strategies.

The use of microfluidic modeling has largely advanced reproductive and IVF-related research, but many studies have yet to include the addition of sperm into female reproductive tract models. In 2024, Yaghoobi et al. published on the use of a female reproductive tract conditioned model to examine sperm separation for superior *in vitro* embryonic development (Yaghoobi et al., 2024). The design consisted of triangular prisms within a PDMS microfluidic with sperm introduced against fluid flow (Yaghoobi et al., 2024). The authors found that sperm samples collected at the highest flow rate were more likely to result in blastocyst formation, proving the model advantageous for IVF quality sperm selection in comparison to traditional IVF sperm centrifugation methods (Yaghoobi et al., 2024). This design demonstrates the use of reproductive microfluidic modeling for possible clinical settings and is a significant leap in future IVF studies.

Described herein are a selection of experiments which work to recreate the reproductive system, placenta, and fetal membranes through models such as EVATAR, VCD-OOC, placenta on-a-chip, and fetal membranes on-a-chip. While there are numerous studies utilizing these 3D platforms, the field is highly specialized and is a unique niche within reproductive studies. These models have proven advantageous as a research tool, and with the passing of the 2023 United States FDA Modernization Act, experimental drugs can now be experimented on non-animal research alternatives including organ on-a-chip modeling (Sunildutt et al., 2023). Organ on-a-chip developers, such as Emulate, have since collaborated with pharmaceutical company Pfizer to design models that can assess drug delivery, therapeutics, and pharmacological damages (Marr et al., 2023). Recently, targets have focused towards reproductive OoC models for preclinical drug trial platforms and for clinical trials on chips (Safarzadeh et al., 2024b; Blumenrath et al., 2020). Replicating reproductive organs proves to be a daunting task, but with the progression of steady work demonstrated by the models described within, the reproductive OoC field has pushed forward. OoC models have proven advantageous for their ability to be diversely fabricated, to study complex transport mechanisms, and to examine reproductive immunology.

## 9 Final remarks on fabrication strategies, transport mechanisms, immunology, and future directions

OoC models are fabricated with a variety of materials and advanced printing techniques. Materials for organotypic platforms are typically polydimethylsiloxane (PDMS), glass, polymethylmethacrylate (PMMA) or polycarbonate (PC) (Cho et al., 2021; Banik et al., 2022; Cameron et al., 2022; Zhang et al., 2022). Reproductive OoC platforms covered within this review were most commonly fabricated with PDMS (Eastman et al., 2022; Gnecco et al., 2018; Richardson et al., 2019; Richardson et al., 2020b; Richardson et al., 2020c; Radnaa et al., 2021; Kim et al., 2022; Ganguly et al., 2021; Mosavati et al., 2020; Pu et al., 2021; Ko et al., 2022; Tantengco et al., 2022; Pemathilaka et al., 2019b; Cho et al., 2021; Gnecco et al., 2017). Other reproductive microfluidic fabrication materials include poly-(ethylene glycol)-dimethacrylate, cyclin olefin polymers, three-lane OrganoPlates, the use of free-standing porous membranes, and solo/duet-microfluidic platforms with pneumatic actuation technology (Schuller et al., 2020; Mandt et al., 2024; Mosavati et al., 2022; Ghorbanpour et al., 2023; Xiao et al., 2017). Advanced printing techniques for many of these models include PDMS-based laser processing, hydrogel-based 3D bioprinting, and UV curable resin-based 3D printing (O’Grady et al., 2021; Leung et al., 2022). After the bulk of the device has been developed, permeable membrane substrates such as Matrigel, gelatin, collagen, and fibronectin are added to mimic *in vivo* conditions (Pu et al., 2021; Biglari et al., 2019; Utagawa et al., 2022; Pitingolo et al., 2019; McClain et al., 2019). The wide variety of fabrication materials and printing techniques allow for diverse OoC designs the can be optimized for any study, specifically aiding biological transport investigations.

OoC models aim to address transport mechanisms and biomarker pathways. Transport mechanisms hold special value in discovering how infection ascends and how inflammatory biomarkers are triggered (Richardson et al., 2020b; Hansen-Pupp et al., 2005; Zhu et al., 2018; Korir et al., 2022). By introducing stressors and toxins, the means at which inflammatory biomarkers travel and the metabolism they undergo can be examined within the microfluidic chambers (Pemathilaka et al., 2019a; Mosavati et al., 2022; Dusza et al., 2023). This guides the study of membrane permeability as metabolites travel across a membrane barrier to the next tissue component (Mandt et al., 2024; Mosavati et al., 2022; Pemathilaka et al., 2019b; Pemathilaka et al., 2022). The reproductive OoC models described herein commonly examine cell invasion, migration, and mechanistic transport from the maternal tissues to the fetal tissues, furthering our understanding of PTB (Pemathilaka et al., 2019a; Schuller et al., 2020; Eastman et al., 2022; Richardson et al., 2019; Richardson et al., 2020c; Richardson et al., 2020b; Radnaa et al., 2021; Kim et al., 2022; Ganguly et al., 2021; Mandt et al., 2024; Mosavati et al., 2020; Mosavati et al., 2022; Pu et al., 2021; Tantengco et al., 2022; Cho et al., 2021; Gnecco et al., 2017). These reproductive transport investigations have proved advantageous in OoC platforms as the resulting immunology can be directly monitored, bridging the gap in biochemical experimentation.

Immune responses due to foreign metabolites are a main component of OoC studies. Immune responses such as enzyme secretion (Cho et al., 2021), macrophage activation (Eastman et al., 2022; Korir et al., 2022), and inflammatory protein release (Richardson et al., 2020b; Radnaa et al., 2021; Ghorbanpour et al., 2023; Xiao et al., 2017; Omere et al., 2020) have all been modeled. Significant biological alterations can result from inflammation and stress activated by foreign metabolites including environmental toxins, cigarette smoke, commensal bacterium, exosomes from infected cells, and nanoparticles (Schuller et al., 2020; Richardson et al., 2019; Kim et al., 2022; Ganguly et al., 2021; Tantengco et al., 2022). Biological alterations like PTB and preeclampsia are medical complications specifically examined in reproductive OoC models (Eastman et al., 2022; Gnecco et al., 2018; Ghorbanpour et al., 2023; Eastman et al., 2021). Immunology studies are of interest in OoC models as the platforms can mimic *in vivo* immune responses activated by unknown metabolites.

Fabrication strategies, transport mechanisms, and immunology studies are key aspects within each work reviewed here. With versatile OoC designs, models can be manipulated to study complex transport mechanisms across different cell compartments, and subsequently, the immune responses to these interactions are directly investigated. Overall, OoC technology is a novel and modern innovation to traditional research modeling that pushes reproductive investigations forward.

The future of OoC modeling is optimistic with avenues for further study in areas ranging from the incorporation of pluripotent stem cells (iPSCs) to full human models (Sunildutt et al., 2023). Each further advancement brings the field closer to the design of full human body-on-chips platforms to simulate the entire body’s drug absorption, distribution, metabolism, and excretion (ADME) (Sunildutt et al., 2023). Currently, organ models are a significant improvement to clinical trials and drug discovery—especially in reproductive research where investigation methods are severely limited (Blumenrath et al., 2020). In the near future, OoC technology could be beneficial for overcoming specific inflammatory conditions including bacterial vaginosis and chorioamnionitis, which currently lack treatment methods that are safe for mothers and growing fetuses (Richardson et al., 2022; Mahajan et al., 2022). The recent OoC developments carve a path for the historical breakthroughs anticipated in succeeding reproductive organ models.

## 10 Conclusion

In this review, we explored fetal membrane and placental biology, OoC history, and recent reproductive OoC designs and advancements. We provided background on human embryogenesis and the supportive function and structure of the fetal membranes and placenta. Reproductive OoC designs encompass fetal membrane models including the FMOC, placental models including the placenta on-a-chip, and full reproductive tract models including the EVATAR. Within these OoC models, we noted the applications they aim to address, such as transport mechanisms and immunology. Advancements to fabrication strategies are highlighted, including the use of modern printing and model designs. Analytical techniques utilized with OoC technology are wide ranging and we provided mass spectrometry, fluorescence microscopy, ELISAs, and impedance spectroscopy examples. Future research opportunities involve overcoming current OoC limitations such as developing universal cell culture medium and reusable materials, while lowering fabrication costs (Mahajan et al., 2022; Farhang Doost and Srivastava, 2024). Ultimately, the future of reproductive OoC modeling consists of collaborating with pharmaceutical companies for drug development, advancing *in vitro* fertilization, and uncovering hidden biological mechanisms which activate preterm birth.

## Figures and Tables

**TABLE 1:** Comparative summary table highlighting model design, study aim, advantages, and limitations of each study within this review.

	Authors	Model and design	Study aim	Advantages	Limitations
Fetal Membrane OoC	Gnecco (Gnecco et al., 2018), Eastman (Eastman et al., 2022), O’Grady (O’Grady et al., 2021)	IFMOC; three chamber PDMS planar device on glass slide	Stimulate infection and monitor biomarker secretion	Allows for the addition of real-time electrochemical signals	Three chamber model without accommodation for amnion layer
	Richardson (Richardson et al., 2019)	AM-OOC; two PDMS chambers connected by microchannels on glass in a microplate	Initiate oxidative stress by cigarette smoke extract and examine inflammatory response	Incorporation of an extracellular matrix, planar model for microscopy imaging	Three chamber model without accommodation for chorion layer or 5 cell types
	Richardson (Richardson et al., 2020c)	FM-OO-C; PDMS platform with porous membrane	Compare membrane permeability of traditional transwell system to two chamber FMOC	FM-OO-C was more sensitive to cell-to-cell crosstalk than transwells	FM-OO-C only incorporates amnion layer and not designed for continuous culture
	Richardson (Richardson et al., 2020b), Radnaa (Radnaa et al., 2021), Kim (Kim et al., 2022), Ganguly (Ganguly et al., 2021)	FMi-OOC; PDMS chambers interconnected through a 24 microchannel array and multiple media reservoirs	Model ascending E. *coli* infection, examine eHMGB1and OATP2B1 transport across the FMi, study response of cadmium exposure	Model incorporates the FMi, a key interest in transport investigations	Omits the addition of macrophage immune cells and omits the investigation of placental infection
	Richardson (Richardson et al., 2022)	FMi-OOC and PLA-OOC; three PDMS chambers interconnected through a 24 microchannel array	Demonstrate statin impact, metabolites, and transport on two individual reproductive interfaces	Model incorporates placental transport of two interfaces	Omits biomechanical stressors and real-time analysis
Placenta OoC	Mandt (Mandt et al., 2024)	Placenta OoC; PEGDMA with glass and PDMS, GelMOD barrier by two-photon polymerization	Examine placental barrier diffusion and membrane barrier coatings	Design with two-photon polymerization allows to precisely mimic placental barrier	Extensive fabrication procedure that could impact biocompatibility if not properly coated
	Pemathilaka (Pemathilaka et al., 2019a)	Placenta OoC; two PDMS layers and a polyester track etched membrane	Examine caffeine transport across the placental barrier	Model incorporates dynamic perfusion	Analysis was conducted off-line and not in real-time
	Mosavati (Mosavati et al., 2020)	Placenta OoC; two PDMS chambers with a polycarbonate membrane	Study the impact of flow rate and membrane porosity on the rate of glucose transfer	Model validated with simulated data and examined physics of flow	Incorporating flow into the model introduced challenges which could impact cell layers
	Mosavati (Mosavati et al., 2022)	Placenta OoC; three lane OrganoPlate	Examine nutrient exchange and glucose transport during infection	Model simulated shear stress with flow	The model membrane consisted of type I collagen which does not precisely mimic the natural membrane
	Ghorbanpour (Ghorbanpour et al., 2023)	Placenta OoC; commercial cyclic olefin polymer with three chambers	Examine placental immune responses and inflammatory proteins during preeclampsia	Investigation demonstrates increased inflammatory cytokines and is an adaptable model	Omits primary placental, trophoblast cells, and immune cells
	Pu (Pu et al., 2021)	Placenta OoC; commercial PDMS model with three chambers	Compare matrix materials and evaluate trophoblast invasion	Best cell adhesion was determined to be fibronectin for future OoC studies	Limited by the number of media channels and invasive cells at seeding
	Ko (Ko et al., 2022)	Placenta OoC; gel patterned model, GelMA with PDMS on glass	Examine cell mobility with changes in oxygen concentration	Model fabricated with a high stiffness material to replicate tissue	Limited by complex fabrication procedure and design
	Schuller (Schuller et al., 2020)	Placenta OoC; PET membranes with interdigitated electrode impedance biosensor	Monitor placental barrier transport of toxic nanoparticles	Model includes embedded impedance biosensor for continuous real-time data collection	TEER extreme sensitivity decreases reproducibility from variation in microchannel size, membrane thickness, and electrode position
	Hori (Hori et al., 2024)	Placental barrier OoC; 3D human trophoblast organoids in column-type model	Examine trophoblast organoid barrier function and transport with TEER	Model incorporates human trophoblast stem cells to form organoids	HUVECs and the syncytiotrophoblast barrier cell layers were not entirely covered, impacting TEER values
Multiple Reproductive OoC	Xiao (Xiao et al., 2017)	EVATAR; quintet, solo, duet microfluidic platforms with multiple pumps and valves	Examine the ovary, uterus, cervix, fallopian tube, and liver during a mimicked 28-day menstrual cycle	Design examines organ-to-organ crosstalk with media perfusion for 28 days	Design is highly complex and does not fully investigate liver metabolic activity and sex hormones
	Tantengco (Tantengco et al., 2022)	VCD-OOC; six interconnected PDMS chambers with microchannel arrays	Investigate exosomes contained inflammation inducing bacteria	Model integrates multiple OoC devices into one working design	Model fails to incorporate immune cells to fully recapitulate the system
	Safarzadeh (Safarzadeh et al., 2024b) Kammala (Kammala et al., 2023)	FMi-PLA-OOC; PDMS chambers with microchannel arrays	Examine drug transfer rate and response to IL-10 engineered extracellular vesicles at both fetal-maternal interfaces	Design allows for the incorporation of seven different cell types to monitor both fetal-maternal interfaces together	Structure of seven cell model is not exactly as seen *in utero*, ethical considerations of obtaining placental tissue, specialized equipment required
	Mahajan (Mahajan et al., 2022)	Vagina OoC; Emulate two chamber PDMS model	Model vaginal microbiome-host interactions and examine barrier integrity and inflammatory cytokines	Model could be advanced into a preclinical model for BV therapy developments	Model fails to incorporate all cell types to fully recapitulate the system interactions
	Izadifar (Izadifar et al., 2024)	Cervix OoC; Emulate two chamber PDMS model	Model cervical microbiome-host interactions and examine barrier integrity and inflammatory cytokines	Experimental model successfully produced on-chip mucus layers	Two chamber models omit many of the layers necessary to monitor all biological mechanisms
	Yaghoobi (Yaghoobi et al., 2024)	Reproductive tract OoC; triangular prisms within a PDMS microfluidic	Examine sperm separation for superior *in vitro* embryonic development	Reproductive OoC models often omit sperm experimentation	Experiment was performed with limited samples and should be expanded to confirm results

## Highlights

- Fetal membrane on-a-chip, placenta on-a-chip, and reproductive model review
- Model fabrications, transport mechanisms, and immune response explanations
- Human embryogenesis and fetal membrane biology overview
- Brief description of organ on-a-chip history and background
